# Extraskeletal osteoma in a canary (*Serinus canaria*)

**Published:** 2017-09-15

**Authors:** Moosa Javdani, Mohammad Hashemnia, Zahra Nikousefat, Mohammad Ghasemi

**Affiliations:** 1 Department of Clinical Sciences, Faculty of Veterinary Medicine, Shahrekord University, Shahrekord, Iran;; 2 Department of Pathobiology, Faculty of Veterinary Medicine, Razi University, Kermanshah, Iran.; 3 Clinical Sciences, Faculty of Veterinary Medicine, Razi University, Kermanshah, Iran.

**Keywords:** Avian, Canary *(Serinus canaria)*, Extraskeletal osteoma, Histopathology

## Abstract

Osteoma is an uncommon bone tumor in avian species and other animals. A 2-year-old male canary (*Serinus canaria*) with a history of an oval mass in the left wing for several months was examined. Radiographs showed a radio-opaque mass. Upon the bird’s owner request, the canary was euthanatized and submitted for necropsy. The histopathologic examination revealed numerous trabeculae consisting of both woven and lamellar bone covered by one to several rows of normal osteoblasts. The trabeculae were closely packed, having only small intertrabecular spaces which contained proliferating osteoblasts, sinusoids and myeloid tissue. Based on clinical, radiographic, and histopathologic findings, the mass was diagnosed as extraskeletal osteoma. To the best of authors’ knowledge, extraskeletal osteoma has not been reported in in avian species so far, and this is the first report of osteoma tumor in the birds. However, benign tumors of bones are extremely rare in the birds, osteoma should be considered as a differential diagnosis in the birds with bone lesions.

## Introduction

Osteoma is a benign slow-growing osteogenic lesion, composed of well-differentiated mature bone tissue, characterized by the proliferation of compact or cancellous bone. A number of authors describe the osteoma as a hamartoma, a benign focal overgrowth of mature cells native to the organ, without the normal architecture of the surrounding tissue, while others consider them as the sclerotic end stage of fibrous dysplasia.^1^^,^^2^

Central, peripheral, and extraskeletal osteoma are three variants of osteoma. Central osteomas arise from endosteum, peripheral osteomas arise from periosteum and extraskeletal soft-tissue osteomas usually develop within a muscle.^3^

These uncommon tumors appear in all domestic species, however, they are more often recognized in horses, cattle, sheep, dogs and cats.^4^^-^^7^ Mandible, maxilla, nasal sinuses, and bones of the face and cranium are the most common sites of involvement in domestic animals.^2^

Bone-forming tumors such as osteomas are extremely rare in free-living and captive birds. In a study of 383 cases of neoplasms in 69 avian species, three cases of osteoma in two domestic ducks (*Anas platyrhynchos*) and a budgerigar were reported (*Melopsittacus undulatus*).^8^ In a 10-year survey, with 2281 necropsy examinations of domestic fowl, only a single case of osteoma was observed.^9^ In a study of 351 cases of tumors in broiler chickens (*Gallus gallus*), only two cases of osteoma were reported.^10^ Hahn *et al*. reported two cases of osteoma in a barred owl (*Strix varia*)^11^ and in 2011, a suspected case of osteoma was described in an eclectus parrot (*Eclectus roratus*).^12^ Cardoso *et al*. observed a case of osteoma in a blue-fronted Amazon parrot (*Amazona aestiva*).^13^ Osteomas are extremely rare tumors in the birds and to the best of our knowledge, the extraskeletal type of osteoma has not been previously reported in avian species. This report describes a case of extraskeletal osteoma in a canary (*Serinus canaria*) for the first time.

## Case Description

**Fig. 1:**
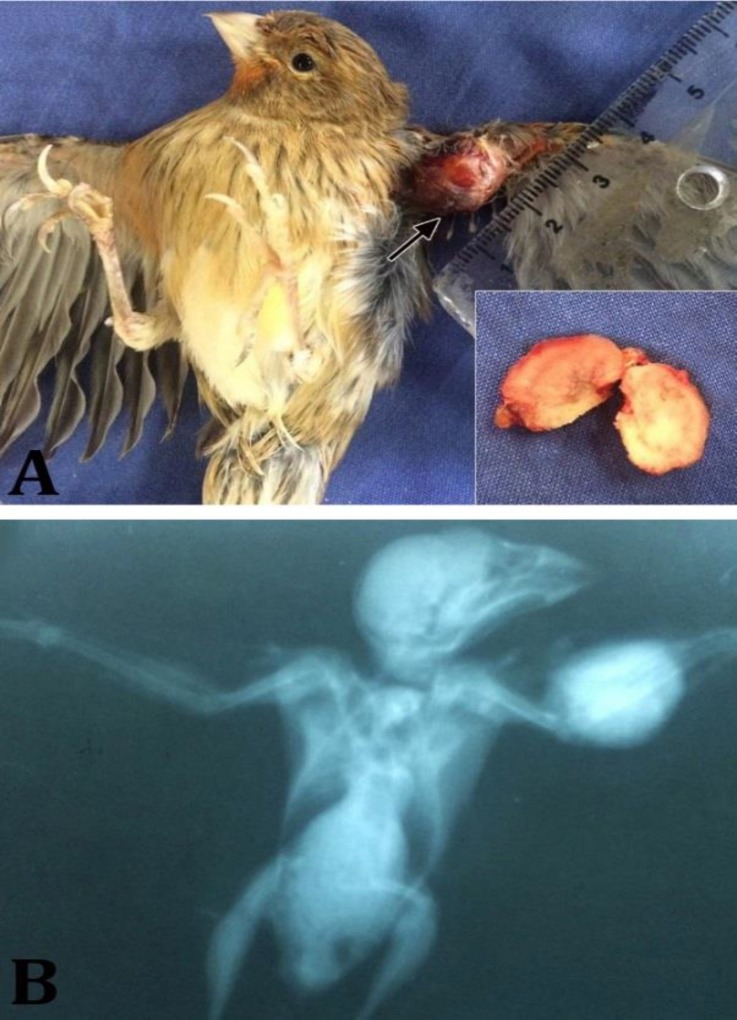
**A)** The encapsulated oval bony mass (arrow) approximately 12 × 8 × 6 mm is observed on the left wing of a *Serinus canaria*; **B)** Radiograph of the left wing shows a regularly ovoid, radio-opaque mass in the forearm region.

A 2-year-old male canary (*Serinus canaria*) was referred to the veterinary teaching Department of Clinical Sciences, Shahrekord University with a history of an oval mass in the left wing, near the forearm region for several months. On physical examination, the mass was firm, well circumscribed, and measured approximately 12×8×6 mm (Fig. 1A). The mass was covered with an ulcerative skin and atrophied muscle. Radiographic evaluation of the bird showed a regularly ovoid, radio-opaque mass in the forearm region (Fig. 1B). Although the surgical intervention was offered, the bird was euthanatized upon the owner’s request. At necropsy, the target mass was located in the soft tissue without any connection to the radius or ulna bones. On transverse section, the mass was whitish, solid and firm in consistency and showed bony structure. For histo-pathologic evaluations, the appropriate tissue samples were fixed in 10% neutral buffered formalin, decalcified in 8% nitric acid, embedded in paraffin, sectioned at 5-µm thickness, and stained with hematoxylin and eosin according to conventional methods.^14^

**Fig. 2:**
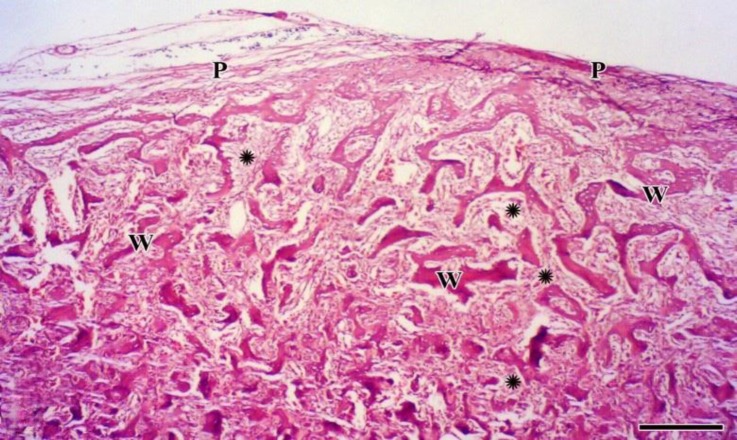
Closely-packed woven bone (W) with small inter-trabecular spaces (stars) surrounded by a periosteum (P), (H & E; Bar = 220 μm

**Fig.3.**
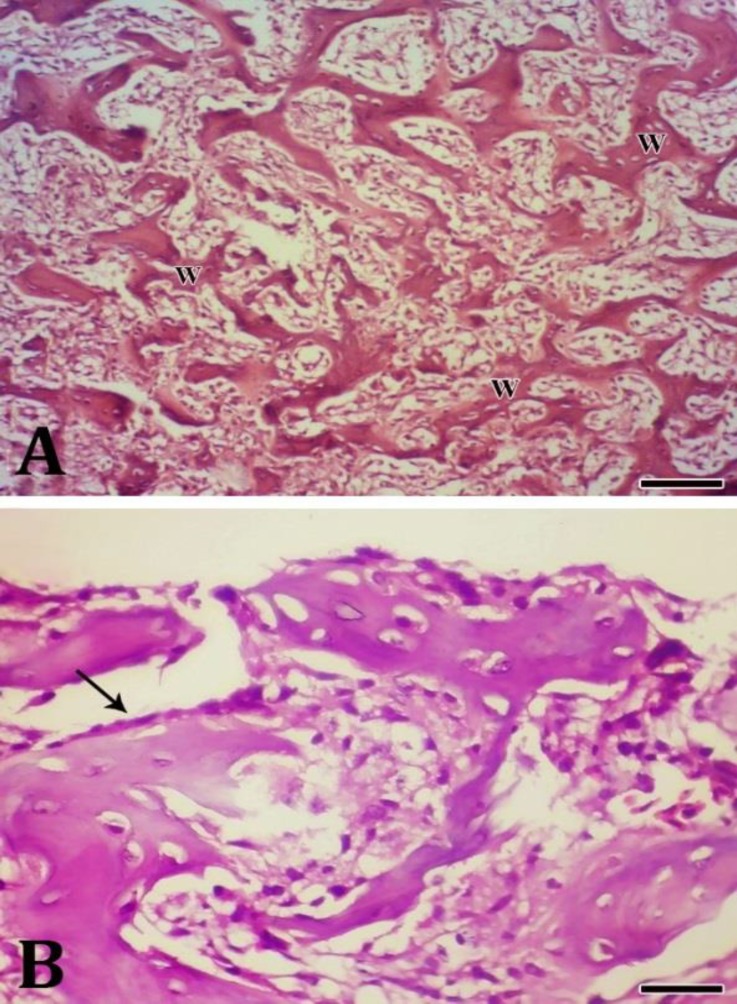
A) Numerous trabeculae consisting of woven bones (W), (H & E; Bar =80 μm); (B) Osseous trabeculae covered by a row of osteoblast with normal appearance (arrows), (H & E; Bar = 20 μm

**Fig. 4:**
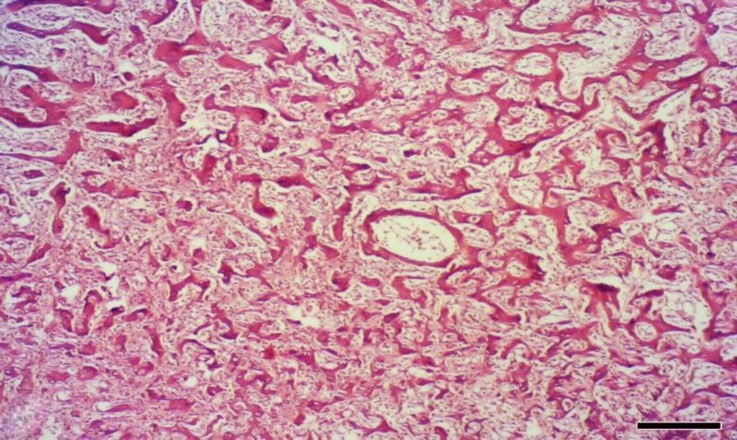
Small intertrabecular spaces contained proliferating osteoblasts, sinusoids and myeloid tissue which varied in amount (H & E; Bar = 220 μm

The histopathologic examination revealed densely woven bone formation as well as the cartilage tissue surrounded by connective tissue. The edge of the mass was appeared smooth and bounded by a periosteum of variable thickness (Fig. 2). Numerous trabeculae consisting of woven bones were observed in the mass. The trabeculae were covered by a row of osteoblast with normal appearance (Figs. 3A and 3B). The trabeculae were closely packed having only small intertrabecular spaces which contained proliferating osteoblasts, sinusoids and myeloid tissue in a different amount (Fig. 4). Based on these findings, the condition was diagnosed as extra-skeletal osteoma.

## Discussion

In this report, we described the clinical and radio-graphic appearance and the pathologic characteristics of extraskeletal osteoma in a canary. To the best of our knowledge, this was the first report describing extra-skeletal osteoma in the birds. 

Benign tumors of bones are not common in animals. Osteoma, ossifying fibroma, and fibrous dysplasia create a group of poorly understood benign lesions arising primarily from membranous bones^2^. Because these tumors are not of great clinical concern or economic significance in the veterinary field, published information that describes these lesions is inadequate. Hence, veterinary pathologists should have information about benign tumors in order to differentiate them from sarcomas of bone.^2^^,^^15^

Osteomas are composed of dense accumulations of well-differentiated cancellous or compact bone with delicate intervening fibrous and vascular tissue. The peripheral trabeculae in osteomas are also immature woven bone, and the older and deeper trabeculae are composed of finely fibered lamellar bone.^2^ Ossifying fibroma is a fibro-osseous lesion that is composed of bone, fibrous tissue, and cementum. Osteoma is composed of dense accumulations of well-differentiated cancellous or compact bone with delicate intervening fibrous and vascular tissues. There are little amounts of bone in ossifying fibromas than in osteomas. Fibrous dysplasia has a major component of fibrovascular stroma separating equidistant, tenuous, curved trabeculae of poorly differentiated bone. These trabeculae arise from metaplasia of fibrous connective tissue and are not typically lined by osteoblasts.^15^^,^^16^

Based on histopathological features, osteomas can be divided into three stages including growing, sclerotic and mature. In growing osteomas, the lesions are surrounded by a dense connective tissue similar to the periosteum, and peripheral trabeculae are formed of woven bone typically deposited by a border of osteoblasts. Sclerotic osteomas are represented by the growth of individual bony trabeculae at the expense of tissue. Mature osteomas are represented by bone fusions of peripheral trabeculae producing a cortical margin of compact bone.^11^^,^^13^

In the present report, the histopathological pattern was similar to the growing osteoma, with a well-formed bone trabeculae bordered by well-differentiated osteoblasts.

Although, trauma, embryonic malformation, infection, developmental disorders, and genetic defects are considered as the etiologic factors for osteoma, however, the pathogenesis of this tumor is poorly understood.^13^ Recently, retroviruses have been shown to be causal agents of some bone tumors in humans and experimental animals, although the etiology is uncertain.^12^ In the present report, there was no evidence of local trauma or infection, therefore the pathogenesis of the osteoma was obscure.

A diagnosis of osteoma is made after considering physical examination, radiographic and histologic findings. In the early stages, the osteoma is often slow growing and asymptomatic and diagnosed incidentally on radiographs, but later on, it can achieve a faster growth rate as the rate of osteogenesis increases and can cause deformation of the bone and compression of the adjacent structures.^11^

Nevertheless, to make a more accurate diagnosis, an immunohistochemical technique (e.g. using SATB2 as a relatively specific immunohistochemical marker of osteoblastic differentiation in bone and soft tissue tumors) can be used.^17^

In conclusion, although, benign tumors of bones are extremely rare in the birds, osteoma should be considered as a differential diagnosis in birds with bone lesions. To make a more accurate diagnosis, the clinical and radio-graphic appearance, as well as the pathologic characteristics of osteoma should be considered.
